# Validity of Pleth Variability Index to Predict Fluid Responsiveness in Patients Undergoing Cervical Spine Surgery in the Modified Prone Position

**DOI:** 10.3390/medicina60122018

**Published:** 2024-12-07

**Authors:** Won Uk Koh, Dong-Ho Lee, Young-Jin Ro, Hee-Sun Park

**Affiliations:** 1 Department of Anesthesiology and Pain Medicine, Asan Medical Center, University of Ulsan College of Medicine, Seoul 05505, Republic of Korea; koh9726@naver.com (W.U.K.); yjro@amc.seoul.kr (Y.-J.R.); 2 Department of Orthopedic Surgery, Asan Medical Center, University of Ulsan College of Medicine, Seoul 05505, Republic of Korea; osdlee@gmail.com

**Keywords:** pleth variability index, prone position, fluid responsiveness

## Abstract

*Background and Objective*: The modified prone position, which is an alteration of the standard prone position, reduces cardiac preload. Dynamic variables including stroke volume variation (SVV), pulse pressure variation (PPV), and pleth variability index (PVI) are reliable predictors for fluid responsiveness during surgery. To the best of our knowledge, no studies assessing dynamic variables for fluid responsiveness have been conducted in the modified prone position. This study aimed to evaluate the ability of PVI to predict fluid responsiveness in the modified prone position during cervical spine surgery. *Materials and Methods:* PVI, SVV, and PPV were recorded at the following times: before and after a 4 mL/kg crystalloid load in the supine position (T1, T2); after placement in the modified prone position (T3); and before and after a 4 mL/kg crystalloid administration in the modified prone position (T4, T5). Fluid responsiveness was defined as stroke volume (SV) ≥ 15%, assessed by the FloTrac/Vigileo™ (Edwards Lifesciences Corp, Irvine, CA, USA). Receiver operating characteristic (ROC) curves were analyzed to identify changes in each dynamic variable that could predict fluid responsiveness in the modified prone position. *Results:* Data from a total of 43 subjects were analyzed. In the supine position, 21 subjects were responders. After subjects were placed in the modified prone position, SV significantly decreased, while PVI, SVV, and PPV significantly increased (*p* < 0.001 for all). In the modified prone position, 13 subjects were responders, and the areas under the ROC curves for ΔPVI, ΔSVV, and ΔPPV after fluid loading were 0.524 (95% confidence interval [CI] 0.329–0.730, *p* = 0.476), 0.749 (95% CI 0.566–0.931, *p* = 0.004), and 0.790 (95% CI 0.641–0.938, *p* < 0.001), respectively. *Conclusions:* Crystalloid pre-loading could not mitigate the decrease in SV caused by the modified prone position. Changes in PVI were less reliable in predicting fluid responsiveness in the modified prone position.

## 1. Introduction

**Figure 1:**
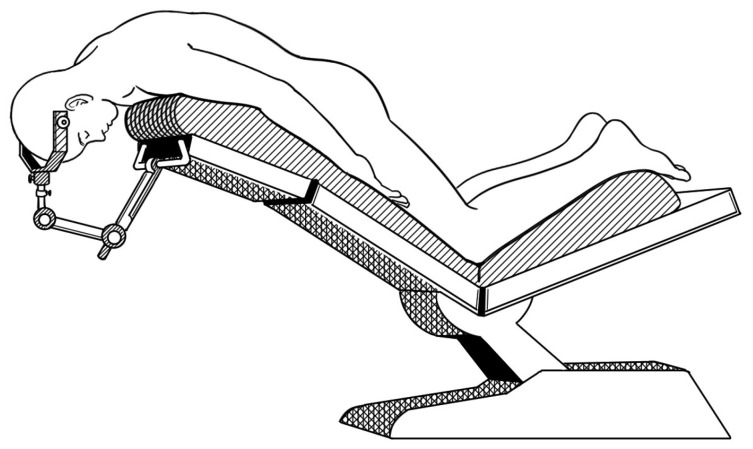
Modified prone positioning, as performed in the current study. The patient is placed in a prone position, with the patient’s head elevated above the level of the heart. Usually, a Wilson frame is placed under the patient’s upper body, but in this figure, the frame is not drawn.

Posterior cervical spine or posterior fossa brain surgery usually requires a modified prone position (Figure 1) or knee-chest position (Concorde position) [1]. This position places the patient in a prone position with a reverse Trendelenburg position to enhance visual exposure of the surgical field through venous drainage. However, it elicits physiological changes such as intravascular blood sequestration in the lower extremities, which can significantly reduce stroke volume, cardiac index, and mean arterial pressure, even affecting the amount of anesthetic agent required [2,3]. Prone position-related hemodynamic compromise can be mitigated by intraoperative fluid administration and the adequate use of vasopressors or chronotropic drugs [4,5]. 

Peri-operative volume expansion has been guided by dynamic variables, which are based on heart–lung interactions during mechanical ventilation. Dynamic variables such as pulse pressure variation (PPV) and stroke volume variation (SVV) have been considered useful tools for assessing fluid responsiveness in the supine [6,7,8,9] and prone positions [10,11,12].

Pleth variability index (PVI) is a non-invasive dynamic variable that measures the respiratory variations of the plethysmographic waveform during mechanical ventilation. PVI has been demonstrated to reliably predict fluid responsiveness in adults undergoing major surgery or intensive care [13,14,15,16]. These studies have predominantly reported on the supine position. However, PVI monitoring is affected by various factors such as high versus low tidal volume, peripheral perfusion, chest wall compliance, and abdominal pressure compared with arterial-derived dynamic variables [17,18,19]. Physiological changes following the prone position may therefore alter the accuracy of dynamic variables in predicting fluid responsiveness, particularly PVI. There are few reports on the validity of PVI for predicting fluid responsiveness in the standard prone position for adults.

Accordingly, this study evaluated PVI, SVV, and PPV to predict fluid responsiveness in the modified prone position. To the best of our knowledge, no studies have evaluated PVI in the modified prone or knee-chest positions.

## 2. Materials and Methods

This prospective study was approved by the institutional review board of the Asan Medical Center (#2018-0340, approval date: 21 March 2018). This study was registered at ClinicalTrials.gov (NCT04002193). We conducted our study in accordance with the Declaration of Helsinki, revised in 2013. Written informed consent was obtained from 50 consecutive patients who were scheduled for elective cervical spine surgery performed in the modified prone position (Figure 1) between December 2019 and September 2020. Patients with American Society of Anesthesiologists physical status I to III, aged 20 years to 80 years, were included. Patients presenting the following were excluded: (1) chronic obstructive pulmonary disease; (2) cardiovascular disease: decompensated heart failure, ejection fraction less than 40% by echocardiography, cardiac arrhythmias such as atrial fibrillation or flutter, and valvular heart disease; (3) moderate-grade pulmonary disease or pulmonary hypertension; (4) body mass index over 30 kg/m^2^ or less than 15 kg/m^2^; and (5) renal disorders (serum creatinine level > 1.5 mg/dl or those with chronic kidney disease).

After patients entered the operating room, an electrocardiographic monitor, pulse oximeter, and non-invasive blood pressure monitor were routinely applied. SEDLine (Masimo Corp., Irvine, CA, USA) monitoring was used to measure the hypnotic effect of anesthetics. A PVI sensor (SET^®^ pulse oximetry sensor, Masimo Corp., USA) was attached to patients before anesthesia induction. The patient was induced with propofol 1.5–2 mg/kg bolus, and total intravenous anesthesia with target-controlled infusion of propofol and remifentanil was administered. A bolus of rocuronium at 0.6 mg/kg was administered to facilitate endotracheal intubation. During anesthesia induction, an arterial catheter was inserted to measure arterial blood pressure. An 18G peripheral line was inserted into one arm for rapid fluid infusion. After induction, somatosensory evoked potential and motor evoked potential monitoring were performed during surgery by a technologist. Muscle relaxation was partially reversed using train-of-four monitoring after the incision. The ventilator was set to volume-controlled ventilation with a tidal volume of 6–8 mL/kg and a respiratory rate of 10–14 breaths/min to maintain ETCO_2_ between 30–35 mmHg and to avoid exceeding a peak airway pressure of 20 cmH_2_O. Positive end expiratory pressure was not applied, and the ventilator settings were not changed during data recording. Cardiac output (CO), stroke volume (SV), and stroke volume variation (SVV) data were measured using a FloTrac/Vigileo™ system (Edwards Lifesciences Corp, Irvine, CA, USA). PPV data were automatically provided by an Intellivue MP80 software system (Philips Healthcare, Amsterdam, The Netherland). The vital signs, patient monitor data (Intellivue MP80), PVI, and FloTrac/Vigileo™ values were recorded using an automatic recording program, Vital Recorder [20].

### 2.1. Study Design and Definition of Fluid Responder

Five minutes after the induction of anesthesia, subjects received a 4 mL/kg of crystalloids (Plasma-Lyte 148^®^, Baxter Incorporated, Seoul, Republic of Korea) over 5–10 min. Measurements were performed before (T1) and 5 min after volume loading (T2). After changing to the modified prone position with the Wilson frame, a third measurement (T3) was recorded when checking the cervical level by the surgeons. Then, the surgical incision was started. When SV was reduced by 15% during the surgical procedure, subjects received a 4 mL/kg of crystalloids over 5–10 min. If SV was not reduced, volume loading was started as decided by the anesthesiologist when the surgical procedure was being conducted under the microscope. These measurements were recorded as before (T4) and 5 min after (T5) fluid infusion in the modified prone position.

During surgery, a bolus of vasopressor such as ephedrine or phenylephrine was used when needed. We waited at least 10 min for stabilization before the initiation of the volume loading, and no vasopressor was administered during data recording. If vasopressors were inevitably used to maintain systolic arterial blood pressure during the recording period, the data were excluded from the study. Responders and non-responders were defined based on changes in SV; following a 4 mL/kg crystalloid challenge in both supine and modified prone positions, responders exhibited an SV change of ≥15%, while non-responders exhibited an SV change of <15%.

### 2.2. Statistical Analysis

A power calculation was performed using Medcalc software (version 17.0.9., Ostend, Belgium). A power calculation showed that a study population of 44 subjects was necessary to demonstrate that PVI can predict fluid responsiveness with an area under the receiver operating characteristics (ROC) curve of 0.88 compared with an area under the null hypothesis of 0.65 (type I error = 0.05, power = 0.9) [10,21]. Allowing for approximately 10% possible dropouts, 50 subjects were included in our study.

Continuous variables are presented as the median (25th–75th interquartile range) for non-normally distributed data or mean (standard deviation) for normally distributed data. Normality was assessed using the Shapiro–Wilk test. Appropriate statistical tests were applied based on the distribution of each variable. The effects of volume loading on hemodynamic parameters were assessed using either the non-parametric Wilcoxon signed-rank test or the parametric paired *t*-test, depending on the normality of the variables. A Bonferroni-adjusted *p*-value was applied to compare variables between T1 vs. T2, T2 vs. T3, and T4 vs. T5 if needed. Assuming that a 15% change in SV was required for clinical significance, subjects were categorized as responders and non-responders based on SV changes of ≥15% and < 15% after the volume challenge, respectively [10]. An ROC curve analysis was conducted to evaluate the predictive abilities of ΔPVI for an increase in SV of ≥ 15% in the modified prone position. The area under the curve (AUC) values of the ROC were calculated. A *p*-value of < 0.05 was considered statistically significant. Statistical analysis was conducted using R version 4.1.2, (https://www.r-project.org/).

## 3. Results

A total of 50 subjects were enrolled in the study; seven were excluded due to surgery cancellation (one), unexpected airway obstruction during surgery (one), and vasoactive or vasodilation drug use during measurements (five). The characteristics of the 43 subjects are shown in Table 1.

Baseline hemodynamic variables and their changes throughout the study are summarized in Table 2. After initial crystalloid loading in the supine position, SV significantly increased (*p* = 0.001), while PVI, SVV, and PPV significantly decreased (*p* = 0.001, 0.001, and 0.024, respectively). When subjects were transferred into the modified prone position, SV and perfusion index significantly decreased, while HR, PVI, SVV, and PPV significantly increased (*p* < 0.001). After the second fluid loading in the modified prone position (T4 vs. T5), SV increased (*p* = 0.074), while PVI, SVV, and PPV decreased. However, these changes in hemodynamic variables were not significant. MAP did not change significantly throughout the study.

The responder and non-responder groups were divided based on a change in SV of ≥15% in the supine position (Table 3). SV significantly increased in responders, while it did not significantly change in non-responders. PVI significantly decreased in responders (*p* = 0.012). SVV significantly decreased in both responders and non-responders (*p* < 0.001 and 0.015, respectively). PPV decreased in responders but was not significant in either group.

In the modified prone position, the changes in hemodynamic variables of responders (n = 13) and non-responders (n = 30) are presented in Table 4. PVI did not significantly change in either responders or non-responders (*p* = 0.409 and 0.074). SVV and PPV significantly decreased in responders (*p* = 0.013 and 0.005, respectively), but not in non-responders.

**Figure 2:**
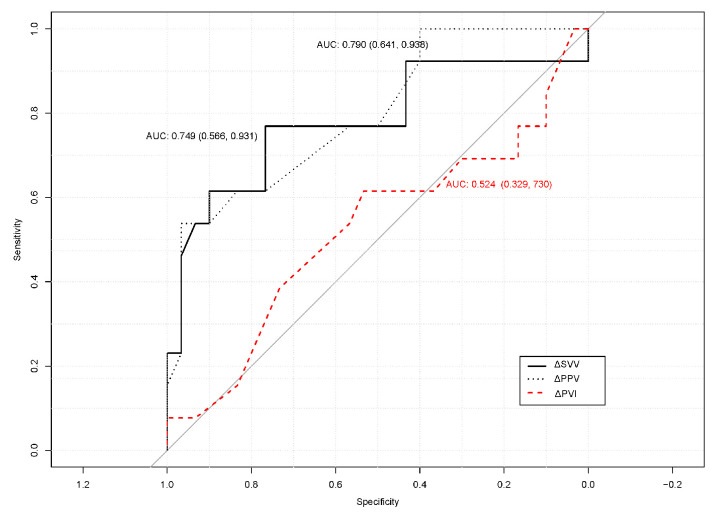
Receiver operating characteristic (ROC) curve demonstrating the ability of Δpleth variability index (PVI), Δstroke volume variation (SVV), and Δpulse pressure variation (PPV) in predicting a stroke volume increase of ≥15%; AUC, area under the curve.

The ROC curve analysis determined the predictive ability of an SV increase of ≥ 15% for the percent change (Δ) of dynamic variables in the modified prone position (Figure 2). The AUCs of ΔPPV and ΔSVV were 0.790 (95% CI 0.641–0.938, *p* < 0.001) and 0.749 (95% CI 0.566–0.931, *p* = 0.004), respectively. However, the AUC of ΔPVI was 0.524 (95% CI 0.329–0.730, *p* = 0.476). In the correlation analysis, ΔPPV (*r* = −0.472 [−0.677, −0.200], *p* = 0.001) and ΔSVV (*r* = −0.561 [−0.737, −0.313], *p* < 0.001) were linearly correlated with ΔSV. However, ΔPVI was not correlated with ΔSV after fluid loading in the modified prone position (*r* = 0.046 [−0.258, 0.342], *p* = 0.770).

## 4. Discussion

This study demonstrated a significant decrease in SV when subjects were placed in the modified prone position despite a 4 mL/kg crystalloid preload before the positional change. In addition, PVI changes showed poor predictive ability for fluid responsiveness compared with SVV and PPV in the modified prone position for cervical spine surgery.

Generally, the prone position and knee-chest positions increase intra-abdominal and intrathoracic pressure, which can reduce venous return, ventricular volume, and, in turn, decrease SV and cardiac output by up to 25–35% [22,23]. The modified prone position, which is altered from the standard prone and knee-chest positions, is also expected to reduce SV and cardiac output. Although the Wilson frame, commonly employed in the prone position, can reduce abdominal and chest compression [5], the modified prone position with head elevation may further aggravate hemodynamic instability. In this study, we observed a significant reduction in SV, and an increase in dynamic variables such as PVI, SVV, and PPV were exhibited, while MAP remained stable.

The ability of dynamic variables can be affected by body position, as this factor can cause physiological changes and modify the extent of cardiopulmonary interaction [12,24,25]. Dynamic variables from arterial pressure monitors, such as SVV and PPV, have been regarded as maintaining the ability to predict fluid responsiveness in the prone position [10,21]; however, there are limited data on their performance in the modified prone position.

The accuracy and reliability of PVI are influenced by various external factors, including the type of surgery, the site of measurement, arterial tone, and peripheral perfusion [19]. There was a report that the application of PVI is more suitable in the absence of elevated intra-abdominal pressure [26]. As PVI is a calculated value from ventilation-induced respiratory changes in the perfusion index (PI) over a constant period, the PI is derived from a pulse oximetry signal and is calculated as the percentage of arterial pulsation (AC) relative to the total amount of light absorbed (DC) in the peripheral tissue [26]. Therefore, factors that affect PI can influence PVI accuracy. In a study where patients received norepinephrine infusion, which acts as a vasoconstrictor, PVI was less reliable than PPV and SVV for predicting fluid responsiveness [27].

Peripheral perfusion is known to affect the reliability of PVI in predicting fluid responsiveness due to its association with PI. Brocher et al. demonstrated that the predictive ability of PVI decreases when PI was < 4% [19]. A previous study found that PI was significantly higher in the supine position compared with the prone and reverse Trendelenburg positions [28]. Our modified prone position requires both arms to be placed straightened and pulled down to expose the cervical spine (Figure 1). Then, the arms are tightly fixed beside the trunk with tape, which can cause compression of the arms and contribute to a reduction in PI. Consequently, PI significantly decreased to below 4% when transitioning from the supine to the modified prone position. This is one of the main reasons that PVI showed insignificant results in the modified prone position. In a previous report, PVI demonstrated predictive ability in the prone position; however, the AUC of the ROC curve for PVI slightly changed compared with the value in the supine position [29]. These results are likely due to positional effects. In the beach chair position, PVI showed predictive ability for fluid loading; however, the AUCs of the ROC curves were smaller than those for SVV and PPV [30]. Although they did not present PI values, it implies that interpreting PVI requires caution when monitoring in specific surgical positions rather than the supine position.

Another factor that induces peripheral vasoconstriction is surgical stress [19]. The incision length of cervical spine surgery is relatively shorter than major open abdominal surgery or multi-level lumbar fusion surgery. The surgical stress of cervical spine operations may be comparable with or relatively lower than that of open abdominal surgeries. However, in the current study, intraoperative somatosensory or motor evoked potential (SEP or MEP) exams were mostly conducted on both upper extremities. These evoked potential (EP) exams involve continuous or intermittent electrical pulse stimulation, which can cause nociceptive irritation during surgery [5] and may contribute to peripheral vasoconstriction, especially when the PVI sensor is attached to a finger. Consequently, EP monitoring may introduce variability and decrease the accuracy of PVI measurement. Further research is therefore needed to assess PVI predictability without EP monitoring in the modified prone position.

The type of fluid administration can affect the reliability of PVI. Studies that used colloids for volume expansion reported more reliable PVI performance in a systematic review and meta-analysis [31]. This is because colloid loading provides better volume expansion, improving macro- and microcirculation [32]. In the current study, crystalloid loading to increase cardiac preload may have been insufficient to produce significant changes. However, because the cervical spine surgery site is a closed area, and tissue edema due to excessive fluid administration could negatively impact surgical outcomes and the airway, we decided to avoid colloid administration in this study.

In previous reports, the PPV cutoff level for fluid responsiveness was not consistent in supine and prone positions [10,21,29]. Similarly, the cutoff value for PVI has been reported to vary widely, ranging from 7 to 20% in a systematic review and meta-analysis [31]. This variability can be attributed to different clinical situations, such as patient positioning, external factors, and varying fluid management protocols across studies. Furthermore, some studies have shown a relatively weak correlation between PVI and other arterial-derived variables [33,34,35]. Therefore, interpreting dynamic variables, especially PVI, requires caution when used in the modified prone position.

Nonetheless, the advantage of PVI is that it provides a non-invasive measurement that can be obtained before arterial line insertion. The percentage change in baseline PVI before and after forced ventilation at the pre-induction stage has been identified as a predictor of anesthesia-induced hypotension [36]. Lee et al. reported that a baseline PVI value of > 16 predicted hypotension after prone position changes [37]. Although further research is required to validate the reliability of baseline PVI as a predictor of hypotension, baseline PVI values can be acquired earlier than arterial-derived dynamic variables, thus allowing for early fluid resuscitation during the induction period of general anesthesia. In addition, the PVI-guided fluid management reduced lactate levels and the volume of crystalloid administered perioperatively in major abdominal surgery compared with the control group [16]. In settings without invasive monitoring, PVI can be effectively used to manage hemodynamic instability and intraoperative fluid management.

Our study has some limitations. The variation in our data was wide in both positions.

Firstly, the measurements in the supine position may have been affected by general anesthesia induction agents. We administered a bolus of propofol and followed by total intravenous anesthesia with propofol and remifentanil. The use of propofol in elderly patients can induce cardiovascular suppression and affect low cardiac output, which may be misinterpreted as being due to low volume status. Secondly, positional changes from supine to prone can strongly stimulate anesthetized patients. Therefore, adjustments to anesthetic drug doses may have been conducted to deepen anesthetic depth. These adjustments may have caused a significant reduction in SV when patients were placed in the modified prone position, even though hemodynamic parameters significantly changed after crystalloid loading. Changes in anesthetic drug infusion doses directly impact hemodynamic variables; this is why the current data showed relatively wide variations. Third, this study analyzed changes in dynamic variables before and after fluid loading to predict fluid responsiveness. However, the number of responders in the modified prone position was small, which may decrease the reliability of PVI for assessing fluid responsiveness. Therefore, future studies may need larger participant groups to accurately assess PVI reliability in the modified prone position. Lastly, the definition of fluid responsiveness varied in previous studies, typically involving a 10–20% increase in CO, CO index, SV, and SV index [12,29,38]. Considering that cervical spine surgery tends to exhibit less hemodynamic variability, the threshold for defining fluid responsiveness in this study might be set lower than 15%.

## 5. Conclusions

PVI monitoring to predict fluid responsiveness is not suitable in the modified prone position. Further research is needed to evaluate PVI reliability in different positions.

## Figures and Tables

**Table 1:** Subjects baseline characteristics (n = 43) and intraoperative variables.

Characteristics	
Demographic data	
Sex (male)	34 (79.1%)
Age (years)	63.8 (9.8)
Weight (kg)	69.5 (9.5)
Height (cm)	166.3 (9.1)
Body mass index (kg/m^2^)	25.1 (2.4)
Comorbidities	
Hypertension	20 (46.5%)
Diabetes mellitus	15 (34.9%)
Intraoperative data	
Anesthesia time (min)	181.8 (31.2)
Crystalloid (mL)	1607.1 (361.4)
Urine output (mL)	155.2 (175.5)
Tidal volume, supine (mL)	454.0 (13.4)
Tidal volume, modified prone (mL)	435.4 (8.6)
Respiratory rate (breaths/min)	11.5 (1.2)
PEEP *	0

Values are mean (standard deviation) or n (%). * PEEP, positive end expiratory pressure.

**Table 2:** Hemodynamic variables at different time points.

	Supine		Modified Prone		
	T1	T2	T3	T4	T5
HR	64 (12)	62 (55–67)	67(10) †	68 (10)	66 (10)
MAP	71 (15)	75 (17)	71 (13)	72 (11)	75 (12)
PVI	14.16 (5.99)	11.56 (5.72) *	20.56 (7.08) †	19.02 (5.49)	17.91 (6.57)
PI	6.4 (4.8–7.5)	5.2 (4.4–7.5)	1.8 (1.1–2.9) †	1.1 (0.9–1.7)	1.1 (0.9–1.7)
SVV	14.1 (3.4)	9.8 (4.1) *	19.7 (5.0) †	20.9 (5.8)	18.9 (6.2)
PPV	9.8 [8.0–14.0]	8.0 [6.0–12.5] *	15.6 [13.0–21.0] †	15.5 [12.0–24.0]	14.0 [10.0–18.5]
SV	65.5 [50.0–68.0]	68.0 [61.0–75.0] *	50.0 [42.5–58.0] †	53.0 [43.0–58.0]	56.0 [47.0–65.0]

Values are the mean (standard deviation) or median [IQR]. T1, before fluid loading; T2, after fluid loading; T3, after placing modified prone position; T4, modified prone position, before fluid loading; T5, modified prone position after fluid loading; HR, heart rate (beats/min); MAP, mean arterial pressure (mmHg); PVI, pleth variability index; PI, pulsatile index; SVV, stroke volume variation; PPV, pulse pressure variation; SV, stroke volume (mL); * *p*-value < 0.05 compared with T1; † *p*-value < 0.05 compared with T2.

**Table 3:** Changes in hemodynamic variables in responders vs. non-responders after fluid loading in the supine position.

	Responders (n =21)					Non-Responders (n = 22)				
	T1	T2	T3	*p*-Value ^1^	*p*-Value ^2^	T1	T2	T3	*p*-Value ^1^	*p*-Value ^2^
HR	68(12)	60 (8)	67 (10)	0.002	<0.001	61(12	63 (12)	62 [58–72]	0.733	0.39
MAP	77 (12)	77 (18)	72 (11)	0.519	0.394	66 (15)	73 (16)	71 (14)	0.122	0.321
PVI	14.4 (7.0)	11.5 (5.5)	21.0 [15.0, 25.0]	0.012	<0.001	13.9 (5.1)	11.7 (6.1)	19.0 [14.3–22.8]	0.122	<0.001
SVV	15.6 (2.3)	9.9 (4.3)	19.3 (4.6)	<0.001	<0.001	12.6 (3.7)	9.7 (4.1)	20.1 (5.4)	0.015	<0.001
PPV	12.0 (4.3)	9.5 (5.1)	17.8 (5.9)	0.057	<0.001	11.4 (4.6)	9.3 (4.5)	17.7 (6.8)	0.079	<0.001
SV	50.0 [43.0–58.0]	66.0 [60.0–72.0]	46.0 [40.0–50.0]	0.000	<0.001	67.5 [62.3–72.0]	69.5 [62.3–75.5]	53.0 [47.3–57.8]	0.696	<0.001

Values are the mean (standard deviation) or median [IQR]. T1, before fluid loading; T2, after fluid loading; T3, after placing modified prone position; HR, heart rate (beats/min); MAP, mean arterial pressure (mmHg); PVI, pleth variability index; SVV, stroke volume variation; PPV, pulse pressure variation; SV, stroke volume (mL); *p*-value ^1^ (T1 vs. T2); *p*-value ^2^ (T2 vs. T3).

**Table 4:** Changes in hemodynamic variables in responders vs. non-responders after fluid loading in the modified prone position.

	Responders (n = 13)			Non-Responders (n = 30)		
	T4	T5	*p*-value	T4	T5	*p*-Value
HR	68 (10)	63 (10)	0.002	68 (11)	67 (10)	0.099
MAP	75 (15)	80 (14)	0.184	71 (10)	72 (10)	0.449
PVI	20.3 (6.4)	19.1 (7.1)	0.409	18.5 (5.1)	17.4 (6.4)	0.074
SVV	23.6 (7.3)	18.5 (8.7)	0.013	19.7 (4.7)	19.1 (4.9)	0.607
PPV	20.5 (7.5)	13.7 (7.1)	0.005	15.8 (7.2)	15.5 (6.4)	0.538
SV	45.7 (13.8)	58.7 (17.9)	0.002	52.9 (10.7)	54.6 (12.1)	0.027

Values are the mean (standard deviation) or median [IQR]. T4, modified prone position, before fluid loading; T5, modified prone position after fluid loading; HR, heart rate (beats/min); MAP, mean arterial pressure (mmHg); PVI, pleth variability index; SVV, stroke volume variation; PPV, pulse pressure variation; SV, stroke volume (mL).

## Data Availability

The data that support the findings of this study are available from the corresponding author upon reasonable request.
